# Doxorubicin cardiomyopathy is ameliorated by acacetin via Sirt1‐mediated activation of AMPK/Nrf2 signal molecules

**DOI:** 10.1111/jcmm.15859

**Published:** 2020-09-11

**Authors:** Wei‐Yin Wu, Yu‐Kai Cui, Yi‐Xiang Hong, Yun‐Da Li, Yao Wu, Gang Li, Gui‐Rong Li, Yan Wang

**Affiliations:** ^1^ Xiamen Cardiovascular Hospital Xiamen University Xiamen China

**Keywords:** acacetin, antioxidant, cardiotoxicity, doxorubicin, oxidative stress, Sirt1

## Abstract

Doxorubicin cardiotoxicity is frequently reported in patients undergoing chemotherapy. The present study investigates whether cardiomyopathy induced by doxorubicin can be improved by the natural flavone acacetin in a mouse model and uncovers the potential molecular mechanism using cultured rat cardiomyoblasts. It was found that the cardiac dysfunction and myocardial fibrosis induced by doxorubicin were significantly improved by acacetin in mice with impaired Nrf2/HO‐1 and Sirt1/pAMPK molecules, which is reversed by acacetin treatment. Doxorubicin decreased cell viability and increased ROS production in rat cardiomyoblasts; these effects are significantly countered by acacetin (0.3‐3 μM) in a concentration‐dependent manner via activating Sirt1/pAMPK signals and enhancing antioxidation (Nrf2/HO‐1 and SOD1/SOD2) and anti‐apoptosis. These protective effects were abolished in cells with silencing Sirt1. The results demonstrate for the first time that doxorubicin cardiotoxicity is antagonized by acacetin via Sirt1‐mediated activation of AMPK/Nrf2 signal molecules, indicating that acacetin may be a drug candidate used clinically for protecting against doxorubicin cardiomyopathy.

## 1 INTRODUCTION

Doxorubicin is an anthracycline chemotherapy drug widely used in clinic for treating breast, endometrial and gastric cancers, childhood solid tumours, soft tissue sarcomas and aggressive lymphoblastic or myeloblastic leukaemia.^1^, ^2^ However, dilated cardiomyopathy and congestive heart failure are frequently reported in patients treated with doxorubicin. Mortality and morbidity are therefore increased when heart failure develops in these patients.^3^, ^4^ Dexrazoxane is the only FDA‐approved drug that is used to protect against doxorubicin‐induced cardiomyopathy,^5^ but it carries the risk potential of increasing secondary malignant neoplasms.^6^ Beta‐adrenoceptor blockers, angiotensin‐converting‐enzyme inhibitors and angiotensin II receptor blockers are reported to be effective in preventing anthracycline‐induced cardiotoxicity^5^; however, the reports from different observations are controversial.^7^ Therefore, new avenues of exploration are needed to develop better pharmacotherapies and interventions to prevent the cardiotoxicity.^8^

It has been reported that several mechanisms are involved in cardiomyopathy induced by doxorubicin, including oxidative stress, mitochondrial dysfunction, calcium overload, apoptosis and disturbance of energy metabolism in cardiomyocytes.^9^ Reactive oxygen species (ROS) are considered to be the main upstream factor in generating myocardial damage by doxorubicin, which generally decreases endogenous antioxidative function and induces cell apoptosis.^10^ In our previous studies, the natural flavone acacetin from the traditional Chinese medicinal herb snow lotus is found to be effective in treating atrial fibrillation.^11^, ^12^ Our recent studies have demonstrated that acacetin confers myocardial protection against ischaemia/reperfusion or hypoxia/reoxygenation injury by inhibiting ROS production, decreasing inflammation and apoptosis via activating antioxidative signalling AMPK/Nrf2 pathway.^13^, ^14^ The present study determined whether acacetin could protect against doxorubicin cardiotoxicity in in vivo moue model and cellular mode with multiple experimental approaches. The results demonstrated that acacetin effectively improved doxorubicin cardiomyopathy by reducing ROS production and apoptosis via Sirt1‐mediated activation of AMPK/Nrf2 signal molecules.

## 2 MATERIALS AND METHODS

### 2.1 Materials

Acacetin (5,7‐dihydroxy‐4‐methoxyflavone) and acacetin prodrug used in this study were synthesized as described previously.^13^, ^14^ Lipofectamine 2000, Annexin V‐APC/SYTOX Apoptosis Detection Kit, 2′7′‐dichlorofluorescein diacetate (DCFH‐DA), Dulbecco's modified Eagle's medium (DMEM) and foetal bovine serum (FBS) were purchased from Thermo Fisher Scientific (Waltham, MA, USA). MTT powder and doxorubicin were purchased from Sigma‐Aldrich (St Louis, MO, USA). Dihydroethidium (DHE) was from Beyotime Biotechnology (Shanghai, China). Masson's Staining Kit and Lactate dehydrogenase assay kit were from Nanjing Jiancheng Bioengineering Institute (Nanjing, Jiangsu, China). Small interfering RNA (siRNA) molecules targeting rat Sirt1 mRNA (sc‐108043) was from Santa Cruz Biotechnology (Dallas, TX, USA), whereas siRNA molecules targeting rat AMPK and scrambled control siRNA were synthesized by RiboBio (Guangzhou, Guangdong, China).

The anti‐Bcl‐2 (sc‐7382), anti‐Bax (sc‐493), anti‐SOD2 (sc‐133134), anti‐AMPK (sc‐25792), anti‐pAMPK (sc‐33524), anti‐LKB1(sc‐32245), anti‐Lamin B1 (sc‐377000), anti‐GAPDH (sc‐20357) and anti‐β‐actin (sc‐130300) antibodies were purchased from Santa Cruz Biotechnology. The anti‐Nrf2 (PB0327), anti‐HO‐1 (PB0212), anti‐SOD1 (BA1401) and anti‐caspase‐3 (BA3257) antibodies were from Boster Biological Technology Co. (Wuhan, Hubei, China), whereas the pLKB1 (#3482) and Sirt1 (#9475) antibodies were obtained from Cell Signaling Technology (Danvers, MA, USA).

### 2.2 Cardiotoxicity induced by doxorubicin

Male C57BL/6 mice (6‐8 weeks) were purchased from Beijing Vital River Laboratory Animal Technology Co. (Beijing, China). The animal experiment protocol was approved by the Animal Care and Ethics Committee of Xiamen University. The animals were cared following the Guide for the Care and Use of Laboratory Animals published by the US National Institutes of Health (NIH Publication No. 85‐23, revised 1996). Mice were randomly assigned to control, doxorubicin and doxorubicin with acacetin treatment groups. Doxorubicin dissolved in saline was administered intraperitoneally at a cumulative dose of 15 mg/kg over a period of 12 days (2.5 mg/kg/day, every other day) followed by 16 days of observation. For the acacetin treatment group, animals were subcutaneously injected with the acacetin prodrug (15 mg/kg, b.i.d.) for 3 days before receiving doxorubicin treatment and then throughout the experimental period in this group of animals. As acacetin prodrug is converted in vivo into acacetin which exerts effective pharmacological actions,^13^, ^15^ the term prodrug is omitted in the following experiments. The animals served for control received equivalent volume of saline. After determining echocardiography, the animals were killed by cervical dislocation at the end of experiment; their hearts were then excised and processed for further histology and biochemical analysis.

### 2.3 Transthoracic echocardiography

*In vivo* heart function was assessed by transthoracic echocardiography in sedated C57BL/6 mice fixed on a heated platform using a Vevo 2100 high‐resolution imaging system (VisualSonics, Toronto, ON, Canada, 40 MHz transducer) at the end of the experiment. Series of M‐mode images at the level of papillary muscles were obtained; left ventricular internal dimensions, left ventricular ejection fraction and left ventricular fraction shortening were measured using at least three consecutive cardiac cycles.

### 2.4 Histopathology

The ventricular tissue was fixed in 4% paraformaldehyde overnight, embedded in paraffin and sectioned into 5‐μm slices. Sections were stained with Masson's trichrome staining kit following manufacturer's instruction. Images were obtained by a microscope equipped with a camera.

### 2.5 TUNEL staining

Myocardial sections were incubated with TUNEL reaction mixture (Cat. No. 11684795910, In Situ Cell Death Detection Kit, Fluorescein, Roche, Mannheim, Germany) for 1 hour at 37°C following the manufacturer's instructions. Subsequently, the sections were washed with PBS and stained with DAPI. Images were obtained by using a confocal microscope (TCS SP5, Leica, Wetzlar, Germany), and the numbers of TUNEL‐positive cells were counted in 5 random fields for each sample.

### 2.6 Measurement of cardiac ROS

ROS level of ventricular tissue was determined in myocardial sections incubated with 10 μM dihydroethidium (DHE) at 37°C for 20 minutes in the dark then washed three times with PBS using a confocal microscope (TCS SP5, Leica) to obtain the fluorescence images which were then quantified by Image J as described previously.^14^

### 2.7 Cell culture and cell viability assay

Rat cardiomyoblasts (H9C2 cells, ATCC, Manassas, Virginia, USA) were maintained in DMEM supplemented with 10% foetal bovine serum, 100 μg/mL streptomycin and 100 units/mL penicillin at 37°C with 5% CO_2_. When cells (passages 5‐10) grew to 70%‐80% confluence, acacetin (0.3, 1 and 3 μM) or vehicle (DMSO) was added for 4‐h incubation. The cells were then exposed to doxorubicin for 24 hours. For viability assays, cells were seeded in 96‐well plates and viability was determined with MTT assay as described previously.^14^ Briefly, the cells were treated with or without doxorubicin in the absence or presence of acacetin, then incubated with 0.5 mg/mL MTT for 4 hours and resuspended in 150 μL of DMSO. Absorbance was measured at 575 nm using an Infinite M200 Pro Nanoquant (TECAN, Switzerland).

### 2.8 Flow cytometry analysis

Cell viability, apoptosis and intracellular ROS level were determined with flow cytometry analysis (Beckman Coulter, USA) as described previously.^16^ Rat cardiomyoblasts were seeded into 6‐well plates and cultured with doxorubicin in the absence or presence of acacetin for cell viability and apoptosis assay. The detached cells were resuspended in binding buffer containing Annexin V and SYTOX, incubated for 15 minutes at room temperature in the dark and then analysed by flow cytometry within 1 hour. ROS level was determined in rat cardiomyoblasts with or without acacetin and subjected to doxorubicin treatment. The cells were then incubated with DCFH‐DA (10 μM) at 37°C for 30 minutes and fluorescence was determined by Flow Cytometer (Beckman Coulter) to obtain ROS levels. Unstable flow cytometry data due to cell population and detachment procedure were discarded from data analysis.

### 2.9 Western blot analysis

The related proteins, that is Nrf2, HO‐1, SOD1, SOD2, Bcl2, Bax, caspase‐3, AMPK, pAMPK, Sirt1, β‐actin or GAPDH, were determined in rat cardiomyoblasts or mouse ventricular tissues by Western blot analysis as described previously.^14^ The samples were lysed by RIPA buffer with protease inhibitors, and the protein concentrations were determined by BCA assay. Equal amounts of total proteins were separated by using SDS‐PAGE and transferred onto PVDF membranes (Bio‐Rad, Hercules, CA, USA) which were blocked by 5% skim milk and incubated with primary antibodies (1:1000) at 4°C overnight. Then, membranes were washed three times with TBST and incubated with secondary antibody (1:10 000) at room temperature for 1 hour. The membranes were visualized by enhanced chemiluminescence (Advansta, Menlo Park, CA, USA) and were exposed to FluoChem E chemiluminescence detection system (ProteinSimple, San Jose, CA, USA). The relative densities of protein bands were analysed by image analysis software. The Western blots used in the results figures are included in Supplemental original data.

### 2.10 RNA interference

The small interference RNAs (siRNAs) were used to silence Nrf2 and Sirt1 in rat cardiomyoblasts as described previously.^16^ Briefly, when cells grew to 60%‐70% confluence, the cells were transfected by siRNA molecules targeting Nrf2 or Sirt1 using Lipofectamine 2000 for 48 hours, and then, the silencing efficiency was determined with Western blot analysis.

### 2.11 RNA isolation and reverse transcription PCR analysis

Total RNA was isolated from cardiomyoblasts using TRIZOL reagent (Invitrogen) and RNA Extraction Kit (Solarbio, Beijing, China) following the manufacturer's protocol. Afterwards, 1 μg of the total RNA was reverse‐transcribed to cDNA by using the Transcriptor First Strand cDNA Synthesis Kit (Roche Life Science), and Quantitative Reverse Transcription PCR was performed by using QuantiTect SYBR Green PCR Kits (Qiagen) on Bio‐Rad CFX96 Real‐Time System. Relative gene expression was normalized to GAPDH. The primer sequences were as follows: Nrf2 (forward, CCTCAGCATGATGGACTTGGA; reverse, TCCTGTTCCTTCTGGAGTTGC), GAPDH (forward, TGACAACTCCCTCAAGATTGTCA; reverse, GGCATGGACTGTGGTCATGA).

### 2.12 Statistical analysis

Data analysis was performed with GraphPad Prism 5.0 (GraphPad Software, Inc, San Diego, CA, USA). Results are presented as mean ± SEM. One‐way ANOVA followed by Tukey's post hoc test was used for comparisons of multiple groups. A value of *P* < 0.05 was considered as statistically significant.

## 3 RESULTS

### 3.1 Protection of acacetin against doxorubicin cardiomyopathy in mice

**FIGURE 1:**
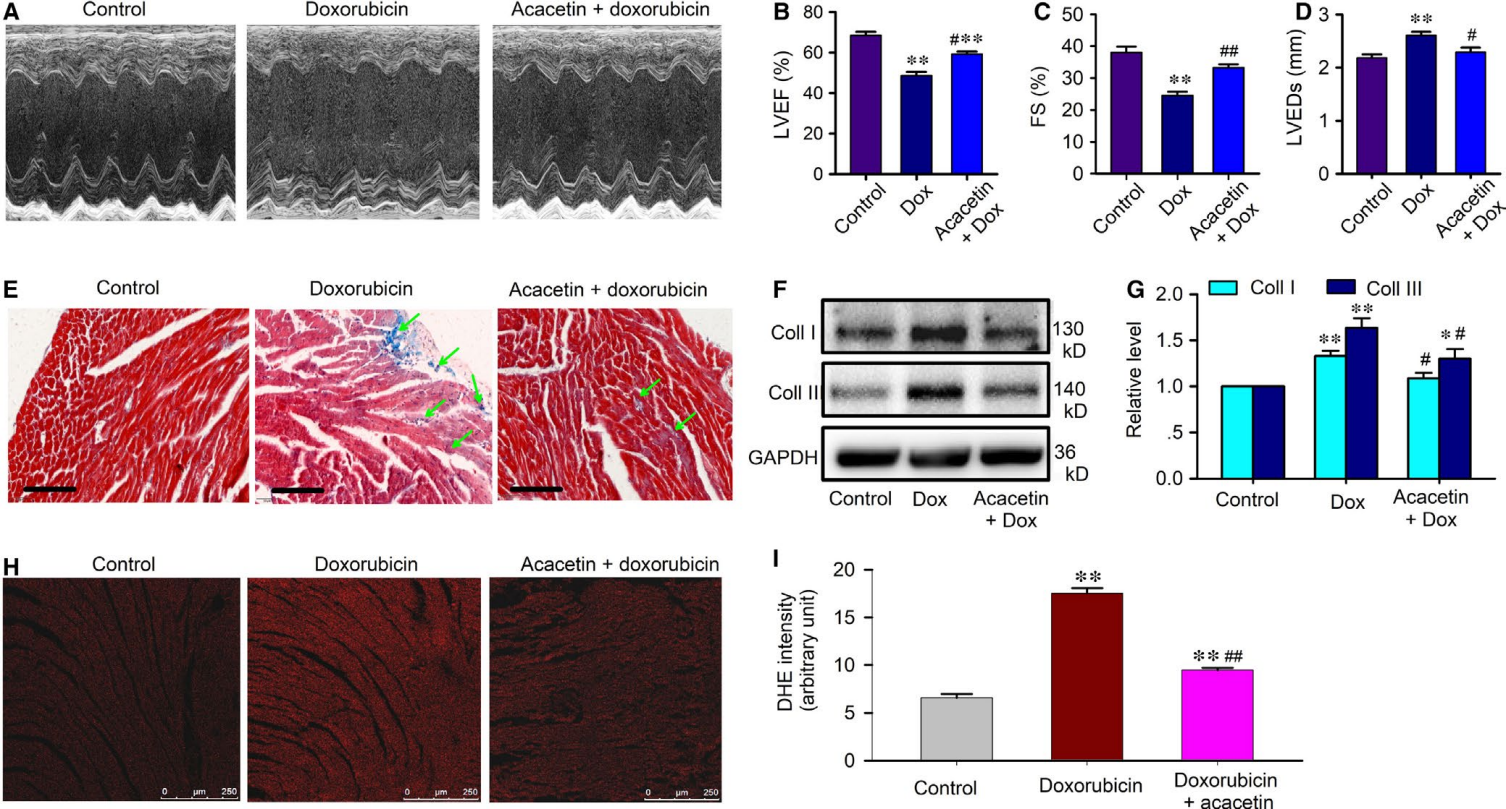
Acacetin prevention of cardiac dysfunction in C57BL/6 mice induced by doxorubicin. A, Representative echocardiographs in C57BL/6 mice treated with vehicle, doxorubicin or acacetin prodrug plus doxorubicin. B, Mean values of left ventricular ejection fraction (LVEF) in mice treated with vehicle, doxorubicin or acacetin prodrug plus doxorubicin. C, Mean values of fractional shortening (FS) in mice treated with vehicle, doxorubicin or acacetin prodrug plus doxorubicin. D, Mean values of left ventricular end‐systolic dimension (LVEDs) in mice treated with vehicle, doxorubicin or acacetin prodrug plus doxorubicin. E, Left ventricular tissue slices stained with Masson's trichrome in mice treated with vehicle, doxorubicin or acacetin prodrug plus doxorubicin. F, Western blots of collagen (Coll) I and collagen III in mice treated with vehicle, doxorubicin or acacetin prodrug plus doxorubicin. G, Mean values of collagen I and collagen III proteins in ventricular tissues of hearts in C57BL/6 mice treated with vehicle, doxorubicin or acacetin prodrug plus doxorubicin. H, Representative photomicrographs showing ROS level as assessed by DHE staining in ventricular tissues of mice treated with vehicle (control), doxorubicin and acacetin prodrug plus doxorubicin. I, Mean values of DHE intensity in ventricular tissues of mice treated with vehicle (control), doxorubicin and acacetin prodrug plus doxorubicin (n = 5, ^**^ *P* < 0.01 vs control; ^##^ *P* < 0.01 vs doxorubicin alone)

In C57BL/6 mice, doxorubicin‐induced cardiomyopathy showed a significant reduction of heart function. Echocardiography (Figure 1A) demonstrated that doxorubicin decreased left ventricular ejection fraction (LVEF, Figure 1B) and fractional shortening (FS, Figure 1C) from 68.4 ± 1.7% and 38.0 ± 1.7% of control (n = 36) to 48.5 ± 1.9% and 24.5 ± 1.5% (n = 24, *P* < 0.01 vs control) and increased left ventricular end‐systolic dimension (LVEDs, Figure 1D) to 2.61 ± 0.07 mm from 2.18 ± 0.07 mm of control (*P* < 0.05). Acacetin treatment significantly reversed the reduction in LVEF and FS to 59.1 ± 1.4% and 33.2 ± 1.1% (n = 28, *P* < 0.01 vs doxorubicin alone) and also reversed the increase in LVEDs to 2.29 ± 0.09 mm (*P* < 0.05 vs doxorubicin alone).

Myocardial histological examination revealed that doxorubicin‐induced increase of ventricular fibrosis was reduced in animals treated with acacetin (Figure 1E). Western blot analysis showed that doxorubicin increased myocardial collagen I and collagen III protein levels (*P* < 0.01 vs control), these increases are significantly inhibited by acacetin (n = 5, *P* < 0.05 vs doxorubicin alone; Figure 1F,G). Moreover, the overproduction of ROS by doxorubicin was observed in myocardium of C57BL/6 mice, which was decreased in animals treated with acacetin (Figure 1H,I).

**FIGURE 2:**
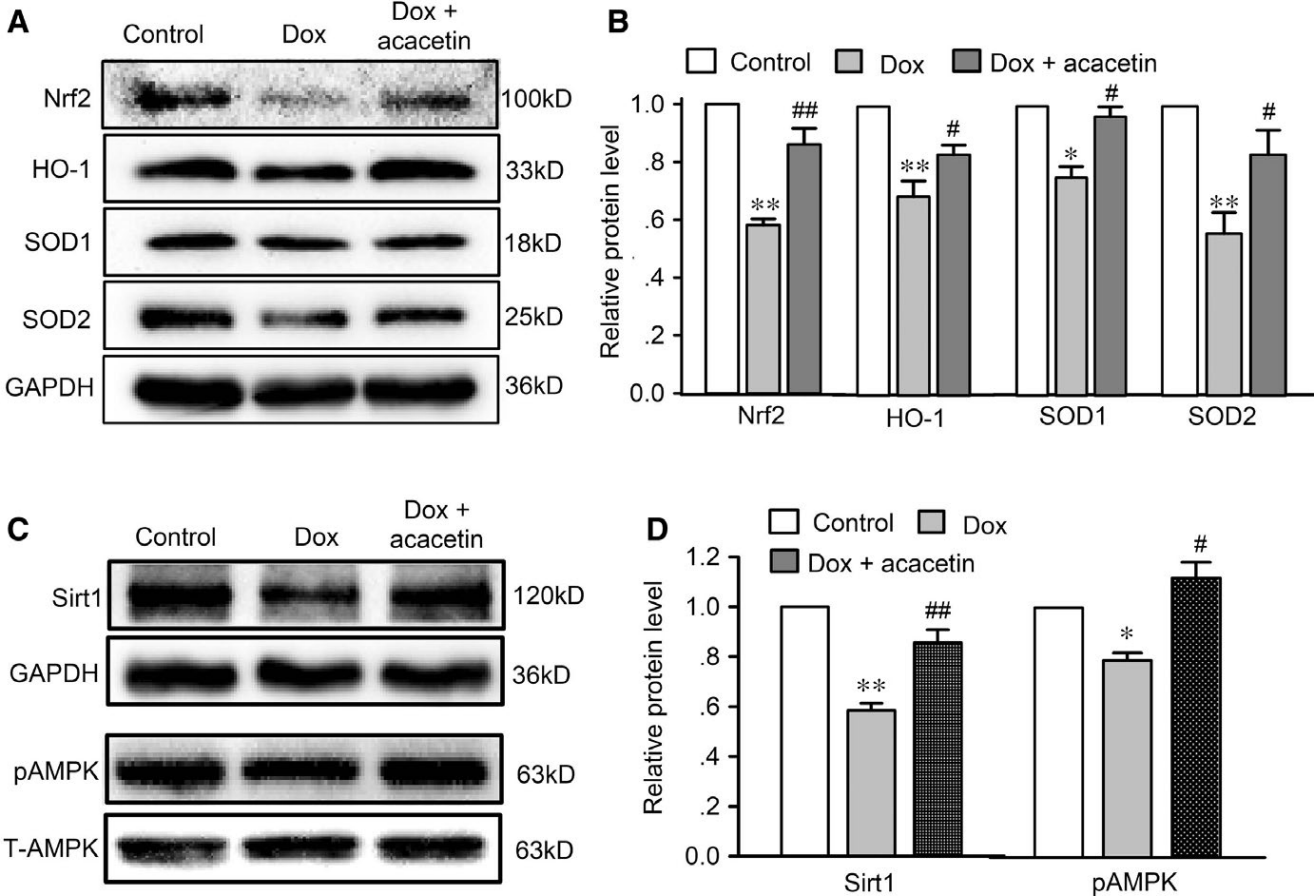
Effects of acacetin on related‐protein expression in ventricular tissues of mice treated with doxorubicin. A, Western blots of Nrf2, HO‐1, SOD1 and SOD2 in ventricular tissues of mice administered with vehicle (Control), doxorubicin (Dox) and acacetin prodrug plus doxorubicin. B, Summarized relative levels of Nrf2, HO‐1, SOD1 and SOD2 expression in ventricular tissues of mice from Western blots as shown in A. C, Western blots of Bcl‐2, Bax and cleaved caspase‐3 in ventricular tissues of mice with the same treatment as in A. C, Western blots and relative level of Sirt1 in ventricular tissues of mice with the same treatment as in A. D, Western blots and relative level of pAMPK and total AMPK (T‐AMPK) in ventricular tissues of mice with the same treatment as in A (n = 5 individual experiments, ^*^ *P* < 0.05, ^**^ *P* < 0.01 vs control; ^#^ *P* < 0.05, ^##^ *P* < 0.01 vs doxorubicin alone)

**FIGURE 3:**
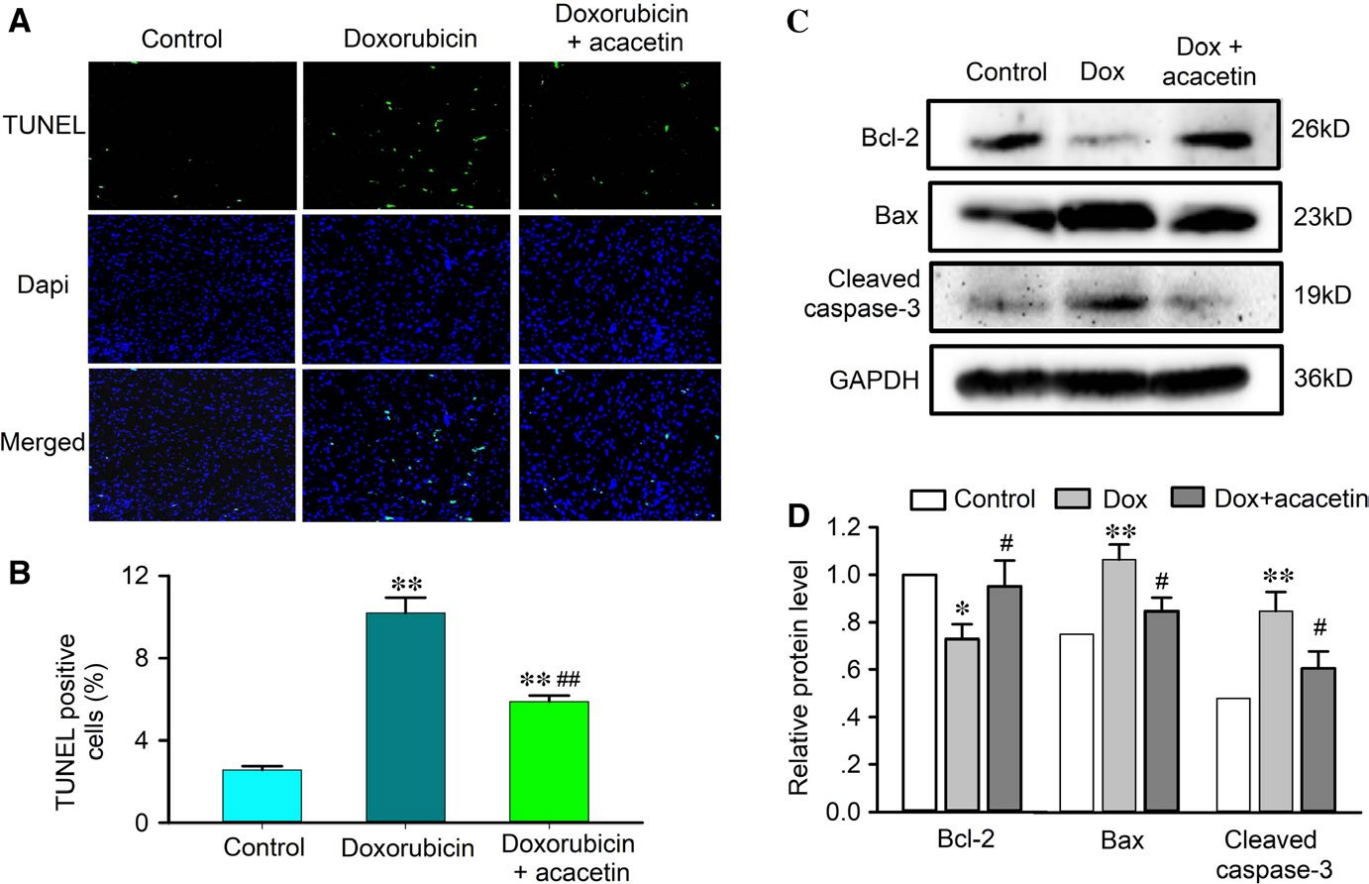
Effect of acacetin on myocardial apoptosis in mouse hearts with doxorubicin. A, Representative ventricular sections stained with TUNEL and Dapi to determine apoptotic cardiomycytes in mice treated with vehicle (control), doxorubicin or doxorubicin plus acacetin. B, Percentage of apoptotic cardiomyocytes in mouse hearts treated with vehicle (control), doxorubicin or doxorubicin plus acacetin. C, Western blots of Bcl‐2, Bax and cleaved caspase‐3 in ventricular tissues of mice administered with vehicle (Control), doxorubicin (Dox) and acacetin prodrug plus doxorubicin. D, Summarized relative levels of Bcl‐2, Bax and cleaved caspase‐3 expression in ventricular tissues of mice from Western blots as shown in C (n = 5 individual experiments, ^*^ *P* < 0.05, ^**^ *P* < 0.01 vs control; ^#^ *P* < 0.05, ^##^ *P* < 0.01 vs doxorubicin alone)

In addition, the myocardial antioxidation proteins (Nrf2, HO‐1, SOD1, SOD2), Sirt1 (a regulator of cellular ageing, apoptosis, stress, etc) and pAMPK (a regulator of cellular energy homeostasis) were down‐regulated in mouse hearts with doxorubicin cardiomyopathy (Figure 2). Myocardial sections stained with TUNEL revealed apoptotic cardiomyocytes were significantly increased in mice with doxorubicin and partially decreased in mice treated with acacetin (Figure 3A,B). The increase of apoptotic cardiomyocytes was associated with reduced anti‐apoptotic protein Bcl‐2 and also with up‐regulated pro‐apoptotic proteins Bax and cleaved caspase‐3 in mice with doxorubicin (Figure 3C,D). Interestingly, acacetin treatment countered the reduction of Nrf2, HO‐1, SOD1, SOD2, Bcl‐2, Sirt1 and pAMPK and the increase of apoptotic cardiomyocytes, Bax and cleaved caspase‐3 (Figures 2 and 3). These results indicate that acacetin significantly protects against doxorubicin cardiotoxicity in mice.

### 3.2 Cellular mechanisms of acacetin protection against doxorubicin cardiotoxicity

**FIGURE 4:**
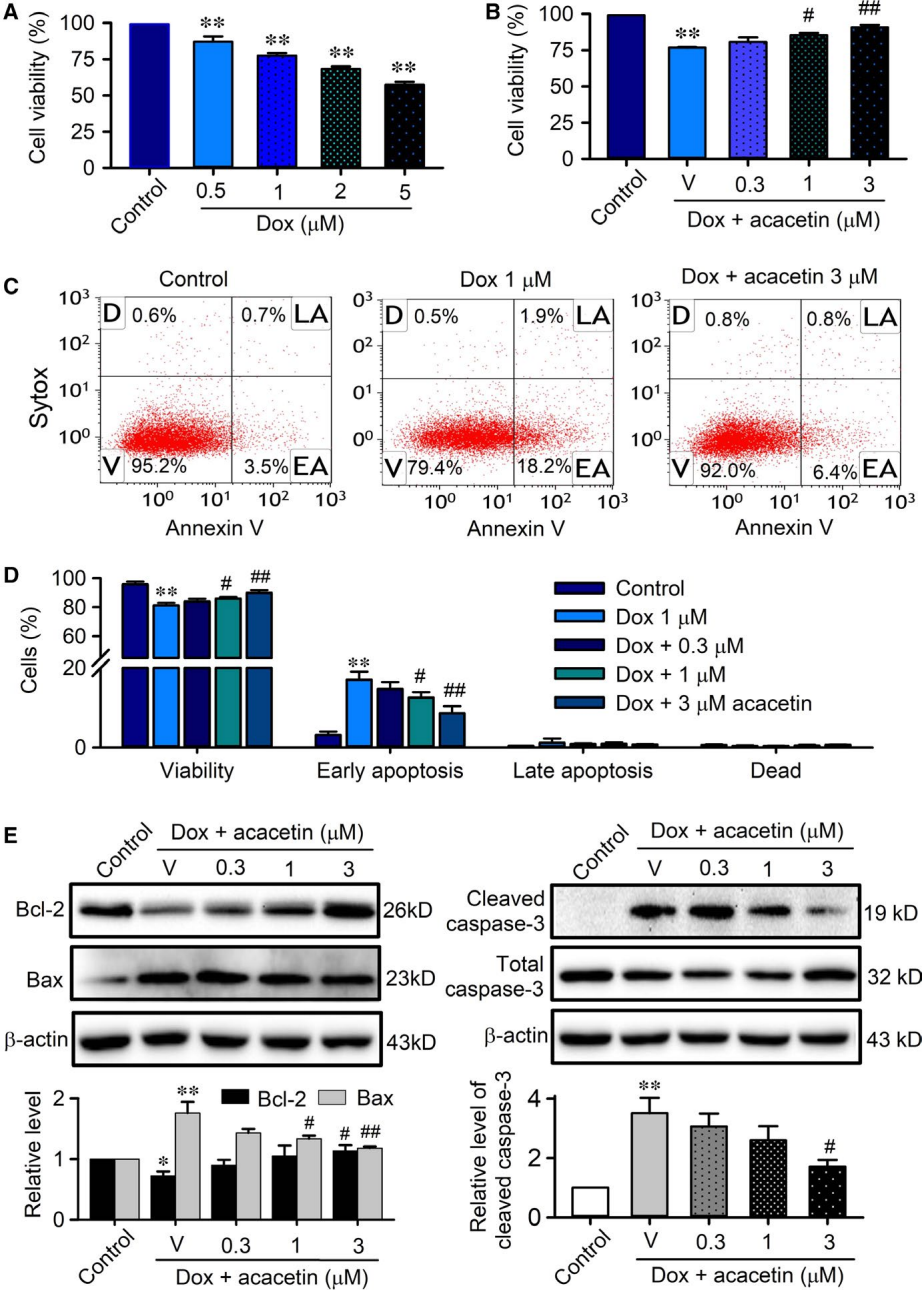
Effects of acacetin on cell viability and apoptosis in rat cardiomyoblasts treated with doxorubicin. A, Cell viability determined by MTT in rat cardiomyoblasts treated with 0, 0.5, 1, 2 or 5 μM of doxorubicin (Dox). B, Cell viability in cardiomyoblasts treated with 1 μM doxorubicin in the absence (V, vehicle) and presence of 0.3, 1 or 3 μM acacetin. C, Flow cytometry graphs show cell viability and apoptosis populations of rat cardiomyoblasts without (control) with 1 μM doxorubicin treatment in the absence or presence of 3 μM acacetin. Cells were labelled with Annexin V‐APC and stained with SYTOX (V, viability; D, dead cells; LA, late apoptosis; EA, early apoptosis). D, Mean per cent values of cell viability, early apoptosis, late apoptosis and dead cells in rat cardiomyoblasts treated with 1 μM doxorubicin in the absence or presence of 0.3, 1 or 3 μM acacetin. E, Western blots and relative mean values of Bcl‐2, Bax and cleaved caspase‐3 in rat cardiomyoblasts treated with 1 μM doxorubicin in the absence or presence of 0.3, 1 or 3 μM acacetin (n = 5 individual experiments, ^*^ *P* < 0.05, ^**^ *P* < 0.01 vs control; ^#^ *P* < 0.05, ^##^ *P* < 0.01 vs doxorubicin alone)

To investigate the potential molecules mediating the protection of acacetin against doxorubicin cardiotoxicity in mice, the effects of acacetin on cell viability and apoptosis were determined in rat cardiomyoblasts subjected to doxorubicin exposure (24 hours). Doxorubicin (0.5‐5 μM) reduced cell viability in a concentration‐dependent manner (Figure 4A). Cell viability was decreased by 1 μM doxorubicin to 77.5 ± 1.7% (n = 5, *P* < 0.01 vs control), and acacetin (0.1‐3 μM) antagonized the viability reduction in a concentration‐dependent manner (Figure 4B).

Flow cytometry analysis revealed that doxorubicin decreased cell viability by promoting apoptosis, which was countered by acacetin (Figure 4C). Acacetin (3 μM) increased cell viability from 81.3 ± 0.7% in cells with doxorubicin to 90.1 ± 0.6% (n = 5, *P < *0.01 vs doxorubicin alone), and the early apoptotic cells were decreased from 17.1 ± 0.8% to 7.6 ± 0.7% (*P* < 0.01 vs doxorubicin alone) (Figure 4D). Western blot analysis revealed that doxorubicin remarkably decreased the anti‐apoptotic protein Bcl‐2 and increased the pro‐apoptotic proteins Bax and cleaved caspase‐3. Acacetin reversed the reduced Bcl‐2 and the up‐regulated Bax and cleaved caspase‐3 in a concentration‐dependent manner (Figure 4E). These results suggest that acacetin significantly antagonizes cell apoptosis induced by doxorubicin.

### 3.3 Molecular mechanisms of acacetin against doxorubicin cardiotoxicity

**FIGURE 5:**
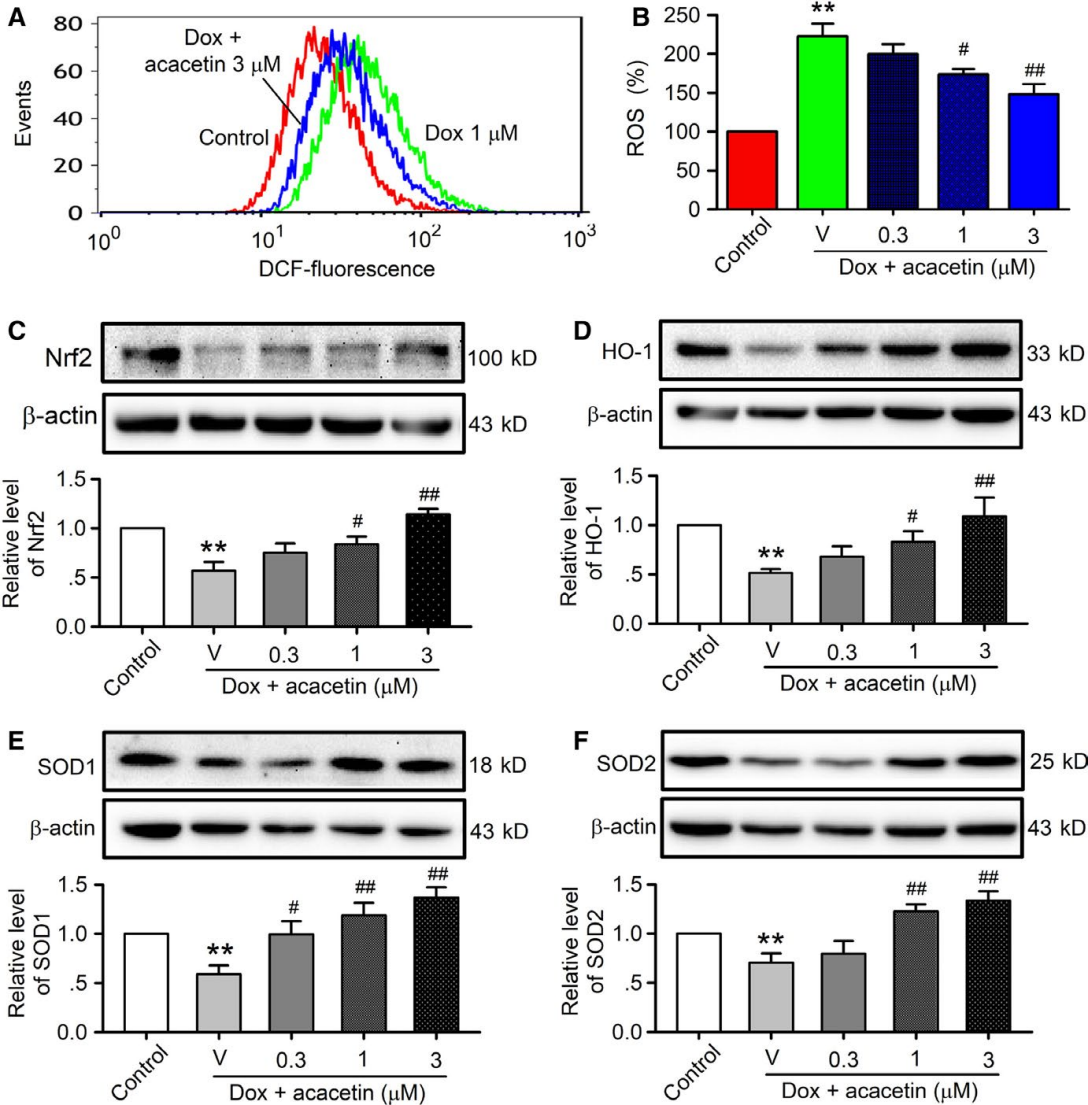
Effects of acacetin on ROS production and antioxidative proteins in cells treated with doxorubicin. A, Flow cytometry graphs showing ROS levels in rat cardiomyoblasts treated without (control) or with 1 μM doxorubicin (Dox) in the absence or presence of 3 μM acacetin. B, Mean per cent values of ROS level in rat cardiomyoblasts without (control) or with doxorubicin exposure in the absence (V, vehicle) or presence of 0.3, 1 or 3 μM acacetin. C‐F, Western blots and mean relative levels of Nrf2 (C), HO‐1 (D), SOD1 (E), SOD2 (F) in rat cardiomyoblasts treated with doxorubicin in the absence and presence of 0.3, 1 or 3 μM acacetin (n = 5 individual experiments, ^**^ *P* < 0.01 vs control; ^#^ *P* < 0.05, ^##^ *P* < 0.01 vs doxorubicin alone)

To determine whether antioxidation is involved in the protective effect of acacetin against doxorubicin cardiotoxicity, ROS production and the proteins related to antioxidation were determined in rat cardiomyoblasts treated with doxorubicin or doxorubicin plus acacetin. ROS level was measured in cells loaded with dichlorofluorescin using flow cytometry. Doxorubicin increased ROS production and acacetin partially reversed this effect (Figure 5A). Acacetin decreased the ROS production induced by doxorubicin in a concentration‐dependent manner (Figure 5B). ROS level was increased to 222.6 ± 16.5% of control by 1 μM doxorubicin and reversed to 148.1 ± 12.9% with 3 μM acacetin (n = 5, *P* < 0.01 vs doxorubicin alone), suggesting that antioxidation is involved in acacetin protection against doxorubicin cardiotoxicity.

It is well known that intracellular ROS level is regulated by endogenous antioxidative system. The effect of acacetin on antioxidation‐related proteins, that is, Nrf2, HO‐1, SOD1 and SOD2, was therefore determined by Western blots in rat cardiomyoblasts treated with doxorubicin or acacetin plus doxorubicin exposure. Doxorubicin remarkably reduced Nrf2 (Figure 5C), HO‐1 (Figure 5D), SOD1 (Figure 5E) and SOD2 (Figure 5F). Acacetin at 0.3, 1 and 3 μM reversed doxorubicin‐induced down‐regulation of these proteins in a concentration‐dependent manner. These results indicate that protection of acacetin against doxorubicin cardiotoxicity is related to limiting ROS production by enhancing antioxidative function in rat cardiomyoblasts.

### 3.4 Nrf2 activation and acacetin protection against doxorubicin cardiotoxicity

**FIGURE 6:**
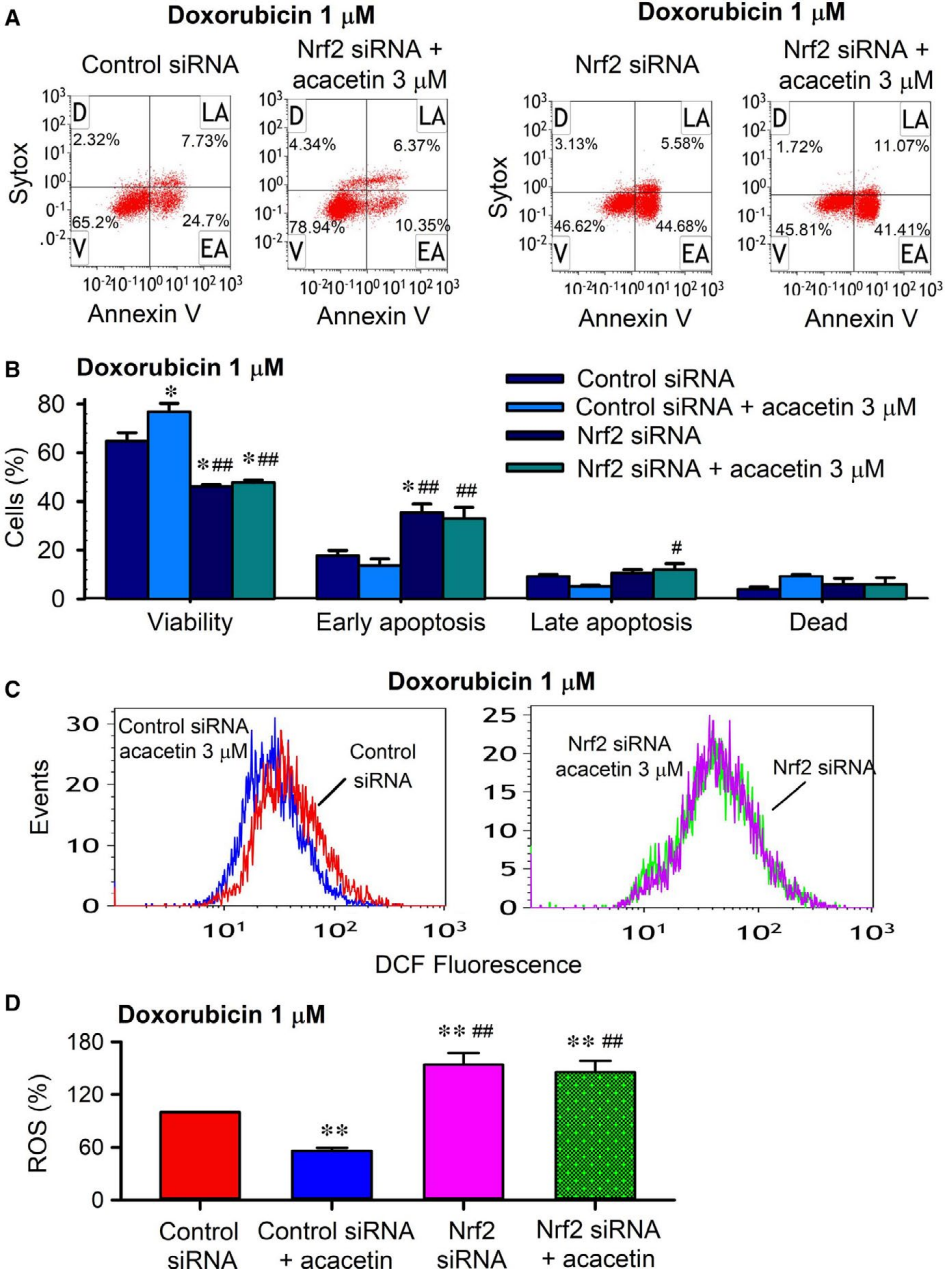
Acacetin protection against doxorubicin cardiotoxicity was abolished in cells with silenced Nrf2. A, Flow cytometry graphs showing cell viability, early apoptosis, late apoptosis and dead cells in rat cardiomyoblasts transfected with control siRNA or Nrf2 siRNA for 48 h and then subjected to 1 μM doxorubicin exposure in the absence or presence of 3 μM acacetin. B, Mean per cent values of cell viability, early apoptosis, late apoptosis and dead cells from flow cytometry graphs as shown in A. C, Flow cytometry graphs showing ROS levels in rat cardiomyoblasts transfected with control siRNA or Nrf2 siRNA and then subjected to 1 μM doxorubicin in the absence or presence of 3 μM acacetin. D, Summarized ROS levels in rat cardiomyoblasts transfected with control siRNA or Nrf2 siRNA from flow cytometry graphs as shown in C (n = 5 individual experiments, ^*^ *P* < 0.05, ^**^ *P* < 0.01 vs control siRNA; ^#^ *P* < 0.05, ^##^ *P* < 0.01 vs control siRNA with acacetin)

It is well recognized that Nrf2 plays a central role in regulating antioxidants in cardioprotection against oxidative stress. To examine whether Nrf2 is involved in protective effect of acacetin against doxorubicin cardiotoxicity, siRNA molecules targeting Nrf2 were transfected into rat cardiomyoblasts. The protective effects of acacetin against doxorubicin cardiotoxicity were then determined in these cells. Acacetin (3 μM) could reverse the cell viability reduction and apoptosis by doxorubicin in cells transfected with control siRNA, but not in cells transfected with Nrf2 siRNA (Figure 6A,B). In addition, acacetin decreased ROS production in cells transfected with control siRNA but not in cells transfected with Nrf2 siRNA (Figure 6C,D). These results suggest that Nrf2 is involved in mediating protection of acacetin against doxorubicin cardiotoxicity.

**FIGURE 7:**
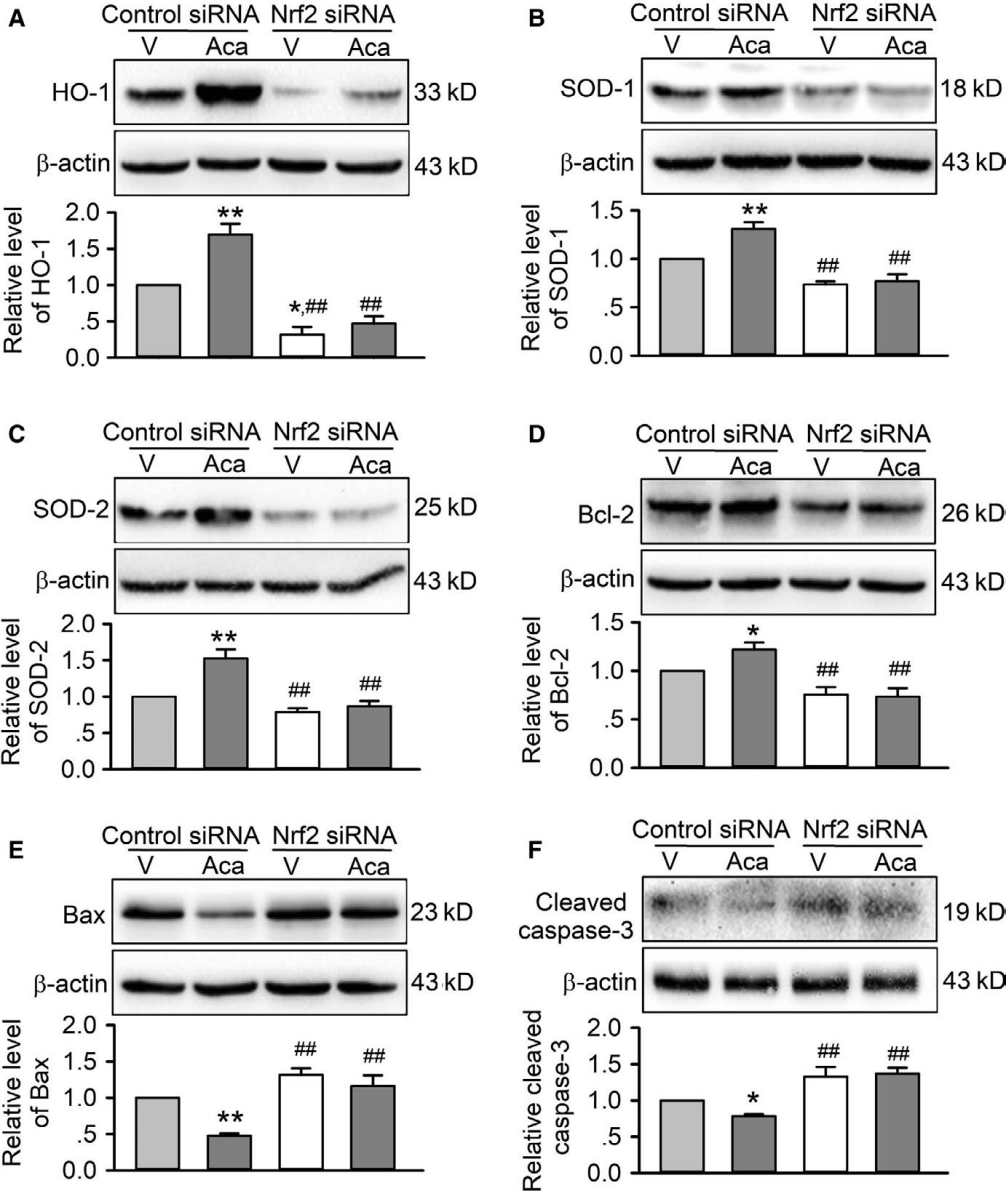
Effects of silencing Nrf2 on antioxidation and apoptosis‐related proteins in cells with doxorubicin exposure. A, Western blots and relative levels of HO‐1 in H9C2 cardiomyoblasts transfected with control siRNA or Nrf2 siRNA and subjected to doxorubicin injury in the absence (V, vehicle) or presence of 3 μM acacetin (Aca). B, Western blots and relative levels of SOD1 in H9C2 cardiomyoblasts with the same treatment as in A. C, Western blots and relative levels of SOD2 in H9C2 cardiomyoblasts with the same treatment as in A. D, Western blots and relative levels of Bcl‐2 in H9C2 cardiomyoblasts with the same treatment as in A. E, Western blots and relative levels of Bax in H9C2 cardiomyoblasts with the same treatment as in A. F, Western blots and relative levels of cleaved caspase‐3 in H9C2 cardiomyoblasts with the same treatment as in A (n = 5 individual experiments, ^*^ *P* < 0.05, ^**^ *P* < 0.01 vs vehicle of control siRNA; ^##^ *P* < 0.01 vs control siRNA with acacetin)

The mediation of Nrf2 for acacetin protection was further demonstrated in following Western blot analysis of the antioxidative enzymes, such as HO‐1, SOD1, SOD2 (Figure 7). Silencing Nrf2 remarkably decreased SOD1 and SOD2, and especially HO‐1 expression, and abolished the increase of these antioxidative proteins by acacetin. In addition, acacetin countered the reduction of anti‐apoptotic protein Bcl‐2 and the increase of pro‐apoptotic factors Bax and cleaved caspase‐3 in cells transfected with control siRNA, but not in cells transfected with Nrf2 siRNA. These results further indicate that antioxidation and anti‐apoptosis properties of acacetin are mediated by Nrf2/HO‐1 signals.

### 3.5 Sirt1 mediates AMPK/Nrf2 activation induced by acacetin

**FIGURE 8:**
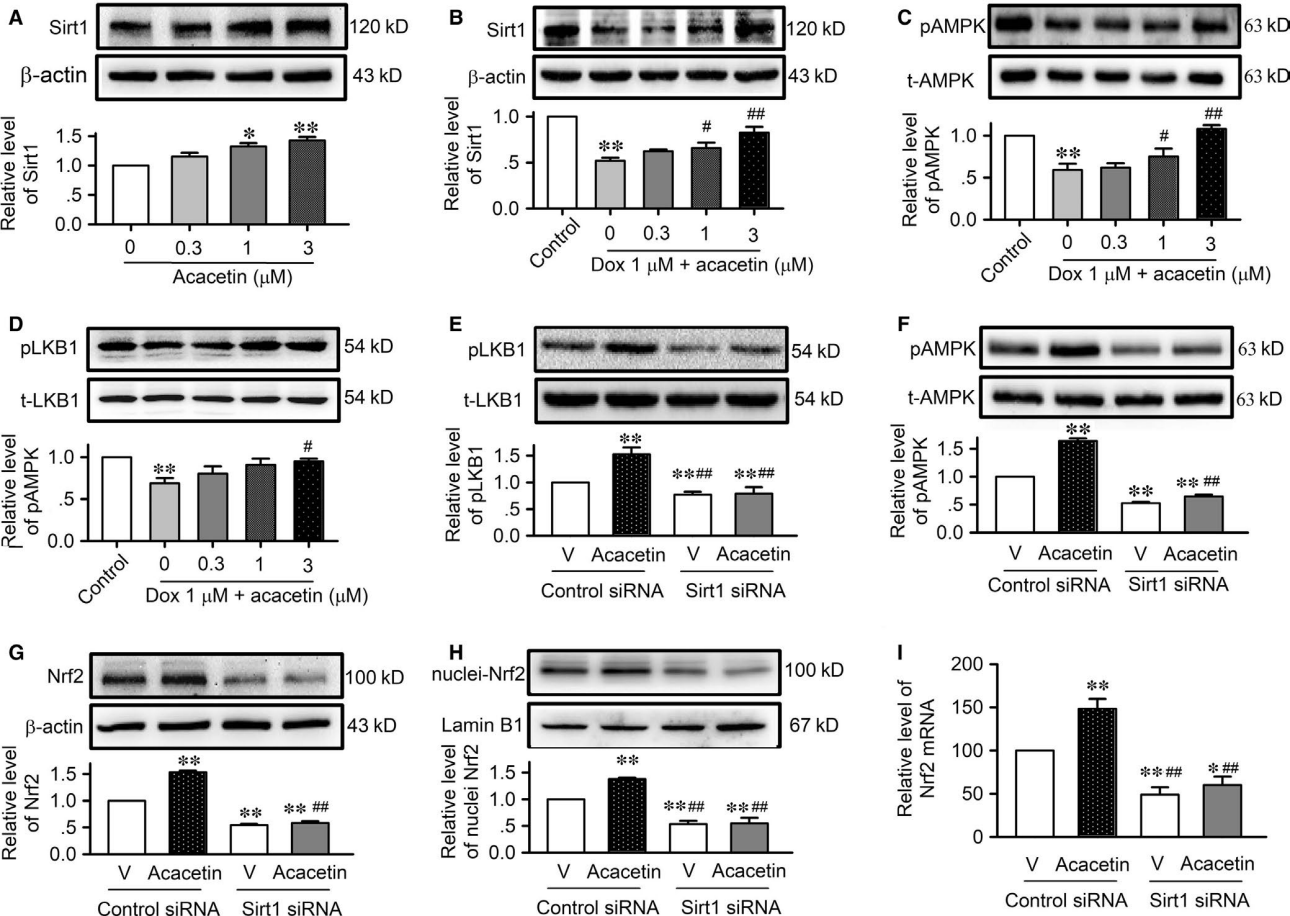
Effects of acacetin on Sirt1 and the dominant signal molecules. A, Western blots and relative level of Sirt1 in rat cardiomyoblasts in the absence and presence of 0.3, 1 or 3 μM acacetin. B, Western blots and mean relative levels of Sirt1 in rat cardiomyoblasts treated with 1 μM doxorubicin in the absence and presence of 0.3, 1 or 3 μM acacetin. C, Western blots and mean relative levels of pAMPK in rat cardiomyoblasts with the same treatment as in B. D, Western blots and mean relative levels of pLKB in rat cardiomyoblasts with the same treatment as in B. E, Western blots and relative level of pLKB and total LKB (t‐LKB) in rat cardiomyoblasts transfected with control siRNA or Sirt1 siRNA in the absence (V, vehicle) or presence of 3 μM acacetin. F, Western blots and relative level of pAMPK and total AMPK (t‐AMPK) in rat cardiomyoblasts transfected with control siRNA or Sirt1 siRNA and treated as in E. G, Western blots and relative level of Nrf2 in rat cardiomyoblasts with the same treatment as in E. H, Western blots and relative level of nuclei Nrf2 in rat cardiomyoblasts with the same treatment as in E. I, Relative Nrf2 mRNA level measured with real‐time PCR in rat cardiomyoblasts with the same treatment as in E. (n = 5 individual experiments, ^*^ *P* < 0.05, ^**^ *P* < 0.01 vs control or vehicle of control siRNA; ^#^ *P* < 0.05, ^##^ *P* < 0.01 vs control siRNA with acacetin)

In rat cardiomyoblasts acacetin increased the relative level of Sirt1 protein in a concentration‐dependent manner (Figure 8A) and countered doxorubicin‐induced reduction of Sirt1 and pAMPK protein expression (Figure 8B,C). It is generally believed that Sirt1 increases pAMPK by activating pLKB1.^17^ It is also the case for acacetin‐induced increase of Sirt1 and pAMPK, because acacetin also reversed doxorubicin‐induced down‐regulation of pLKB1 in a concentration‐dependent manner (Figure 8D). Whether Sirt1 mediates the acacetin‐induced activation of pLKB1, pAMPK and Nrf2 was further determined in rat cardiomyoblasts transfected with siRNA molecules targeting Sirt1. Silencing Sirt1 did not affect total LBK1 and AMPK expression, but decreased pLBK1 and pAMPK and abolished acacetin‐induced enhancement of pLBK1 (Figure 8E) and pAMPK (Figure 8F). Moreover, silencing Sirt1 also significantly decreased the expression of both whole‐cell Nrf2 (Figure 8G) and nuclei Nrf2 (Figure 8H) and abolished acacetin‐induced increase of both whole‐cell Nrf2 (Figure 8G) and nuclei Nrf2 (Figure 8H). The alteration of Nrf2 was correlated to its mRNA expression (Figure 8I). These results demonstrate that doxorubicin cardiotoxicity is related to the overproduction of ROS and inhibition of Sirt1, thereby leading the reduction of antioxidation and increased myocardial apoptosis. Acacetin protects against doxorubicin cardiotoxicity by increasing Sirt1 and the Sirt1‐dependent activation of pAMPK, the antioxidative regulator Nrf2 and the antioxidative proteins, thereby decreasing ROS production, and inhibiting apoptosis. All the findings support the notion that protection of acacetin against doxorubicin cardiotoxicity is mediated by Sirt1‐dependent regulation of AMPK/Nrf2 signal molecules.

## 4 DISCUSSION

Doxorubicin and its derivative epirubicin are widely used anthracyclines to treat breast, endometrial and gastric cancers, childhood solid tumours, soft tissue sarcomas, and aggressive lymphoblastic or myeloblastic leukaemia.^18^ However, the use of anthracyclines is associated with dose‐dependent cardiotoxicity.^4^ Doxorubicin cardiotoxicity is manifested as arrhythmias, ischaemia, systolic dysfunction, due to cardiac cell death and necrosis.^19^ Although dexrazoxane is effective in antagonizing doxorubicin cardiomyopathy,^5^ the potential of increasing secondary malignant neoplasms is reported.^6^ The present study demonstrates that acacetin is very effective in protecting against doxorubicin cardiotoxicity.

Acacetin is a natural flavone compound that exists widely in plant pigments. We have previously reported that acacetin isolated from the traditional Chinese medicinal herb snow lotus possesses unique effects of preferentially inhibiting atrial potassium channels including I_Kur_ (ultra‐rapidly activating delayed rectifier potassium current), I_K.ACh_ (acetylcholine‐activated potassium current), I_to_ (transient outward potassium current) and SK_Ca_ current (small conductance Ca^2+^‐activated potassium current), which contribute to its selective anti‐atrial fibrillation properties.^11^, ^15^, ^20^ Studies from other groups demonstrated that extensive beneficial effects of flavonoids were related to the scavenging free radicals and antioxidant effects.^21^, ^22^ However, free radical scavenging activities of flavonoids depend on benzene ring hydroxyl group and number of their chemical structure.^21^, ^22^, ^23^ No free radical scavenging activity is observed for acacetin,^21^ because of its structure lacks a hydroxyl group in its B‐ring.

Other reports showed that acacetin has anti‐inflammation, anti‐tumour and antioxidative properties.^24^, ^25^ Our recent studies reported that acacetin and its water‐soluble prodrug confer cardioprotection against hypoxia/reoxygenation insult in cardiomyocytes^14^ and ischaemia/reperfusion injury in in vivo and ex vivo hearts.^13^ Acacetin prodrug significantly improves myocardial function and inhibits ventricular arrhythmia induced by ischaemia/reperfusion injury in rats; acacetin prevents ischaemia/reperfusion by increasing myocardial antioxidation and inhibiting inflammation and apoptosis.^13^ Cellular experiments reveal that AMPK‐mediated Nrf2/HO‐1 activation is involved in myocardial protection through multiple effects including antioxidation, anti‐inflammation and anti‐apoptosis.^14^ The present study showed that acacetin significantly improved doxorubicin‐induced cardiac dysfunction and cardiotoxicity via reducing oxidative stress by Sirt1‐dependent activation of AMPK/Nrf2 signals.

It is well recognized that endogenous antioxidant defensive system has evolved in aerobic organisms including human beings to counteract the harmful effects of free radicals and reactive oxygen species (ROS) and maintain redox homeostasis.^26^ ROS, that is, peroxides, superoxide, hydroxyl radical and singlet oxygen, are produced intracellularly through multiple mechanisms depending on the cell and tissue types in cell membranes, mitochondria, peroxisomes and endoplasmic reticulum.^27^ From a biological context, ROS are formed as a natural by‐product of the normal metabolism of oxygen and have important roles in cell signalling and homeostasis. However, during times of environmental stress (oxidative stress), ROS levels can increase dramatically, which may result in significant damage to cell structures by inducing a longer opening of mitochondrial permeability transition pore.^28^ Oxidative stress condition can be caused by either increased ROS formation, decreased activity of antioxidants or both in aerobic organisms. Moderate oxidative stress causes cell dysfunction, whereas excessive oxidative stress usually leads to cell death. For instance, during cardiac ischaemia/reperfusion overwhelming ROS production exceeds the scavenging activity of endogenous antioxidants, thereby leading to cell inflammation, apoptosis and heart dysfunction.^29^ Oxidative stress is generally believed to be the primary cause of doxorubicin cardiotoxicity.^2^ ROS overproduction impairs the antioxidative regulator Nrf2 which causes down‐regulation of HO‐1, NQO‐1, SOD, GST, etc, and promotion of cardiomyocytes apoptosis by decreasing the anti‐apoptotic protein Bcl‐2 and increasing the pro‐apoptotic proteins Bax and cleaved caspase‐3.^30^ The present study showed that acacetin reversed doxorubicin‐induced Nrf2 reduction which significantly increased HO‐1, SOD1 and SOD2, decreased ROS production, elevated Bcl‐2 level, and reduced Bax and cleaved caspase‐3 expression. These effects may play an important role in preventing doxorubicin‐induced cardiotoxicity and cardiac dysfunction.

Our recent study showed that Nrf2 activation by acacetin is dependent on AMPK activation.^14^ An earlier study reported that inhibition of AMPK by doxorubicin accentuated genotoxic stress and cell death, whereas activation of AMPK markedly reduced the effects of doxorubicin in mouse embryonic fibroblasts and cardiomyocytes,^31^ which indicate that AMPK plays a crucial role in decreasing oxidative stress and cell death induced by doxorubicin. The present study demonstrated additional novel information that increase of pAMPK by acacetin is dependent on Sirt1 activation. Silencing Sirt1 reduced pAMPK and abolished the pAMPK increase by acacetin, thereby decreased Nrf2 expression and Nrf2 activation by acacetin in cardiomyoblasts. The notion that Sirt1 mediates AMPK phosphorylation by activating pLKB1 is supported by previous in vivo and ex vivo studies on resveratrol.^32^, ^33^, ^34^

It is clear that Sirt1‐dependent activation of AMPK/Nrf2 signals is involved in prevention of cardiotoxicity by acacetin. In C57BL/6 mouse animal model, Sirt1, pAMPK, Nrf2, and also HO‐1, SOD1, SOD2, Bcl‐2 are remarkably down‐regulated in ventricular tissues by doxorubicin and are recovered by acacetin. The animals treated with acacetin showed an improved heart function with reduced myocardial fibrosis. These results indicate that acacetin is very effective in preventing the cardiotoxicity and cardiac dysfunction induced by doxorubicin.

In addition to the cardioprotective effects observed in the present and previous studies, acacetin has been found to have anti‐cancer effects by suppressing the invasion and migration of human cancer cells. Acacetin inhibits the proliferative activity of human non‐small‐cell lung cancer A549 cell line by promoting p53 and Fas/FasL apoptotic system,^35^ decreases the TPA‐induced adhesion, invasion, and migration of A549 cancer cells by inactivating JNK signalling pathway and reducing binding activities of NF‐kappaB and AP‐1,^25^ and inhibits the invasion and migration of DU145 cells by suppressing p38 MAPK signal pathway.^36^ Acacetin was found to induce Bax activation and mitochondrial damage‐mediated apoptosis in Jurkat T cells^37^, ^38^ and also show selective anti‐cancer activity against chronic lymphocytic leukaemia.^39^ Doxorubicin is widely used in the treatment of lung cancer, breast cancer, prostate cancer, leukaemia, etc.^1^, ^2^, ^9^ Interestingly, a recent study showed that acacetin enhances the therapeutic efficacy of doxorubicin in non‐small‐cell lung carcinoma cells.^40^ Therefore, in addition to the protection against doxorubicin cardiotoxicity, acacetin may have synergetic effect with doxorubicin for treating cancers.

Several natural active compounds, for example dioscin, sulforaphane, resveratrol and quercetin, have been reported also to confer protection against doxorubicin cardiotoxicity by reducing ROS production and regulating apoptosis‐related proteins via activating Sirt1,^32^ Nrf2,^41^ Bmi‐1 expression^42^ or adjusting microRNA‐140‐5p.^43^ Although these natural compounds show promising therapeutic potential, their low solubility and low bioavailability are barriers for drug development. Acacetin is a novel Sirt1 activator that mediates activation of AMPK/Nrf2 signals. The Sirt1 activation by acacetin (unpublished observation) is similar to resveratrol (related to increasing nicotinamide adenine dinucleotide‐dependent deacetylases and nicotinamide phosphoribosyltransferase).^34^ Importantly, the water‐soluble prodrug of acacetin y^13^, ^15^ allows it to be administered intravenously with doxorubicin to prevent cardiotoxicity in patients undergoing chemotherapy in the near future.

In the present study, myocardial fibrosis and collagen proteins were remarkably increased in mice with doxorubicin, which were significantly reversed in animals treated with acacetin. The limitation was that the potential mechanism of anti‐fibrotic effect of acacetin was not explored in the present study, which remains clarified in the future study. However, this would not affect the conclusion that acacetin confers the protection against doxorubicin cardiotoxicity.

Collectively, the present study demonstrates for the first time that acacetin prevents the cardiotoxicity and cardiac dysfunction induced by doxorubicin by enhancing endogenous antioxidants (HO‐1, SOD1 and SOD2) and down‐regulating cardiomyocyte apoptosis via Sirt1‐dependent activation of AMPK/Nrf2 signals. The Sirt1‐mediated activation of AMPK/Nrf2 signal molecules by acacetin plays an important role in rescuing and antagonizing myocardial injury induced by the anti‐cancer drug doxorubicin, which indicates that this natural flavone is likely a promising drug candidate for preventing the cardiotoxicity in patients undergoing doxorubicin chemotherapy.

## CONFLICT OF INTEREST

The authors declare no conflict of interest.

## AUTHOR CONTRIBUTION

**Wei‐Yin Wu:** Conceptualization (lead); Data curation (lead); Formal analysis (lead); Funding acquisition (lead); Investigation (lead); Methodology (lead); Software (lead); Validation (lead); Writing‐original draft (lead); Writing‐review & editing (equal). **Yu‐Kai Cui:** Data curation (supporting); Investigation (supporting); Methodology (supporting); Software (supporting); Validation (supporting); Visualization (supporting). **Yi‐Xiang Hong:** Data curation (supporting); Investigation (supporting); Methodology (supporting); Software (supporting). **Yun‐Da Li:** Data curation (supporting); Investigation (supporting); Methodology (supporting); Software (supporting). **Yao Wu:** Conceptualization (supporting); Data curation (supporting); Methodology (supporting). **Gang Li:** Conceptualization (supporting); Methodology (supporting); Resources (supporting); Validation (supporting). **Gui‐Rong Li:** Conceptualization (lead); Data curation (lead); Formal analysis (lead); Methodology (lead); Project administration (lead); Resources (equal); Supervision (lead); Validation (lead); Writing‐original draft (equal); Writing‐review & editing (lead). **Yan Wang:** Conceptualization (lead); Data curation (lead); Funding acquisition (lead); Methodology (equal); Project administration (lead); Resources (lead); Supervision (lead).
